# Artificial Intelligence in Neurology: A Critical Narrative Review of Clinical Evidence, Translation, and Responsible Implementation

**DOI:** 10.1002/brb3.71873

**Published:** 2026-11-06

**Authors:** Anwar Zahran, Raya Fuqha, Aya Milhem, Orabi Hajjeh, Hossam Salameh, Mohammed AbuBaha, Waseem Shehadeh, Hammam Jallad, Mohammad Bdair, Issa Snoubar, Omar Abu‐Khazneh, Bara AbuBaha, Ameer Awashra, Aseel Eid, Ahmad Mohammad, Ahmad R. Awwad

**Affiliations:** ^1^ Department of Medicine Faculty of Medicine and Health Sciences An‐Najah National University Nablus Palestine; ^2^ Hurley Medical Center‐MSU Flint Michigan USA; ^3^ Department of Neurology Mercy St. Vincent Medical Center Toledo Ohio USA

**Keywords:** artificial intelligence, clinical translation, electrophysiology, external validation, neuroimaging, neurology, responsible AI

## Abstract

**Purpose:**

Artificial intelligence (AI) is increasingly applied to neuroimaging, electrophysiology, clinical text, and digitally measured behavior, but reported performance often exceeds the maturity of clinical evidence.

**Method:**

We conducted a structured narrative review, informed by SANRA principles, of English‐language human studies, reviews, and regulatory sources identified in PubMed/MEDLINE and IEEE Xplore from January 2022 through July 2026; older landmark studies were retained when necessary for historical or methodological context. We applied a common translational‐evidence framework across six prespecified neurological domains to distinguish technical performance from external validation, prospective clinical evaluation, implementation, and demonstrated patient benefit. Evidence was appraised according to cohort representativeness, patient‐level data separation, external and prospective validation, calibration, clinical comparators, regulatory status, workflow integration, and patient‐level benefit.

**Finding:**

Across stroke, dementia, Parkinson disease, epilepsy, multiple sclerosis, and neuro‐oncology, the strongest evidence concerns narrowly defined imaging and signal‐processing tasks. A cluster‐randomized stroke study demonstrated shorter thrombectomy workflow time, and several externally validated models show improved portability. In contrast, many high‐accuracy classification and segmentation studies remain retrospective, single‐dataset, internally validated, and uncalibrated. Multimodal learning, foundation models, federated learning, and large language models broaden capability but introduce additional risks related to dataset shift, bias, opacity, privacy, and automation.

**Conclusion:**

Neuro‐AI should be judged by its intended clinical task and evidence maturity rather than headline accuracy. Multicenter external validation, prospective workflow evaluation, calibration, subgroup reporting, human‐factors testing, and post‐deployment surveillance are prerequisites for safe and equitable clinical use.

## Graphical abstract

A structured narrative review across six neurological domains finds that AI can support imaging, electrophysiology, prognosis, monitoring, and workflow tasks, but clinical evidence remains uneven. Selected external and prospective studies show promise, including faster stroke thrombectomy workflow, while many high‐accuracy models remain retrospective and single‐dataset. Safe use requires external validation, calibration, prospective workflow evaluation, subgroup assessment, human oversight, and lifecycle monitoring; benchmark accuracy alone does not establish patient benefit.

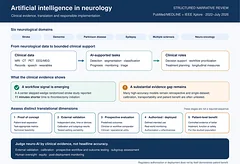

## 1 Introduction

Neurological disorders are a leading cause of global disability and mortality, creating substantial diagnostic, therapeutic, and health‐system burdens (GBD 2021 Nervous System Disorders Collaborators 2024; World Health Organization 2024; R. Yang et al. 2025; GBD 2021 Stroke Risk Factor Collaborators 2024). Their clinical heterogeneity and dependence on complex longitudinal data make neurology an important setting for evaluating AI, while also making overgeneralization especially hazardous.

The burden is unequally distributed. Dementia alone affects tens of millions of people (Alzheimer's Disease International 2023), while many low‐ and middle‐income settings face severe shortages of neurologists, advanced imaging, electrophysiology services, and longitudinal follow‐up (Uwishema and Boon 2025). AI could extend specialist capacity, but only if models are validated in the populations, devices, and workflows in which they will be used.

Neurological practice generates high‐dimensional data from magnetic resonance imaging (MRI), computed tomography (CT), positron emission tomography (PET), electroencephalography (EEG), magnetoencephalography (MEG), genomics, laboratory biomarkers, electronic health records, speech, video, and wearable sensors. These inputs create opportunities for earlier detection, quantitative monitoring, prognosis, and treatment support, but their variability across centers also creates substantial dataset shift.

Terminology requires precision. Artificial intelligence (AI) is the umbrella term; machine learning (ML) denotes algorithms that learn patterns from data, and deep learning (DL) is a subset of ML based on multilayer neural networks. Automated signal processing does not necessarily use ML. Wearables are data‐acquisition platforms, digital biomarkers are objectively measured indicators derived from digital data, and closed‐loop devices are therapeutic systems that may or may not contain adaptive ML. These categories are related but not interchangeable (Topol 2019; Hirani et al. 2024; Miotto et al. 2017).

The clinically relevant question is therefore not whether an algorithm performs well on a benchmark, but whether it improves decisions or outcomes in a defined target population. Internal cross‐validation, external validation, prospective workflow evaluation, regulatory authorization, deployment, and demonstrated patient benefit represent distinct translational stages and should not be discussed as equivalent forms of evidence (US Food and Drug Administration 2025; Amaro 2022).

Several recent reviews provide broad overviews for AI concepts, human–AI interaction, and learning health systems in neurology (Rizzo and Dawson 2025; Rizzo 2025a, 2025b), while disease‐specific reviews address individual applications such as stroke triage, Alzheimer disease (AD) biomarkers, or seizure detection (Heo 2025; D. Peng et al. 2025). The novelty of the present review is therefore not the subject of neurological AI itself but the application of a common clinical‐evidence framework across six prespecified neurological domains. Rather than comparing algorithms primarily by reported accuracy, we examine how far representative applications have progressed from technical proof of concept toward external validation, prospective clinical evaluation, real‐world implementation, and demonstrated patient benefit. This cross‐domain approach shifts attention from the algorithm to the clinical claim and the evidence supporting it, while separately considering calibration, comparators, study quality, regulation, workflow integration, and implementation.

The scope is intentionally selective rather than encyclopedic. We examine stroke, dementia and AD, Parkinson disease (PD) and movement disorders, epilepsy, multiple sclerosis (MS), and neuro‐oncology, together with cross‐cutting developments in multimodal learning, foundation models, large language models (LLMs), FL, explainability, and governance. Headache, neuromuscular disease, neurocritical care, traumatic brain injury, infectious and autoimmune neurology, pediatric neurology, sleep medicine, and neurogenetics merit dedicated reviews and are outside the disease‐specific scope of this article.

## 2 Methods

### 2.1 Review Design and Information Sources

This article is a structured narrative review rather than a systematic review or meta‐analysis. Its design and reporting were informed by the Scale for the Assessment of Narrative Review Articles (SANRA) (Baethge et al. 2019). PubMed/MEDLINE and IEEE Xplore were searched for English‐language publications dated from January 1, 2022, through July 31, 2026; the final electronic searches and targeted update were completed on August 7, 2026. The 2022 start date was chosen a priori to foreground contemporary model classes, validation practices, and regulatory expectations within a defined, clinically interpretable window. Earlier reports were retained only when needed to establish a clinical tool, historical benchmark, or methodological concept. Complete database‐specific search strings are reproduced in Supporting Information Method S1. Regulatory and governance information was obtained from primary sources including the US Food and Drug Administration, the European Union, the World Health Organization, and the International Medical Device Regulators Forum. Reference lists of key reviews and primary studies were also examined.

### 2.2 Search Execution and Study‐Selection Workflow

Searches combined terms for AI, ML, DL, neural networks, LLMs, foundation models, FL, explainable AI, radiomics, and digital biomarkers with terms for the prespecified neurological domains, data modalities, and clinical tasks. Candidate records were reviewed first by title and abstract and then in full text when potentially eligible. The review did not use independent duplicate screening, and disagreements were not recorded in a formal screening log.

Duplicate and overlapping reports were identified using title, digital object identifier, cohort, and system name. When several reports described the same cohort or system, the most complete or clinically mature report was prioritized. Because study identification was purposive rather than exhaustive, a PRISMA‐style count of retrieved and excluded records was not prospectively maintained and is not retrospectively reconstructed. This workflow is reported explicitly to avoid implying systematic coverage.

### 2.3 Eligibility Criteria

Eligible evidence included human primary studies, systematic or scoping reviews, consensus or reporting guidance, regulatory documents, and clinically relevant technical benchmarks. We excluded non‐neurological applications, animal‐only work without a direct clinical‐translation objective, duplicate or superseded reports, and reports with insufficient methodological information for interpretation. Selection favored studies that clarified evidence maturity, external validity, clinical comparison, deployment, or patient‐level utility rather than studies selected solely because they reported the highest performance.

### 2.4 Data Extraction, Evidence Prioritization, and Synthesis

For representative studies, we extracted the clinical task, population and sample size, dataset or input modality, model family, validation design, patient‐level separation, external‐validation status, comparator, reported performance, calibration or confidence intervals when available, explainability method, regulatory or deployment status, and principal limitations. Table 2 provides a concise comparison, and the complete field‐level extraction is reported in Table S1. Interpretation emphasized class balance, external and prospective validation, subgroup performance, clinician comparison, workflow effect, and patient‐level outcomes. Appraisal was informed by Transparent Reporting of a multivariable prediction model for Individual Prognosis Or Diagnosis + Artificial Intelligence (TRIPOD+AI), Prediction model Risk Of Bias ASsessment Tool + Artificial Intelligence (PROBAST+AI), Standards for Reporting of Diagnostic Accuracy Studies‐Artificial Intelligence (STARD‐AI), Checklist for Artificial Intelligence in Medical Imaging (CLAIM), Developmental and Exploratory Clinical Investigations of DEcision support systems driven by Artificial Intelligence (DECIDE‐AI), Standard Protocol Items: Recommendations for Interventional Trials‐Artificial Intelligence (SPIRIT‐AI), and Consolidated Standards of Reporting Trials‐Artificial Intelligence (CONSORT‐AI). (Vasey et al. 2022; Rivera et al. 2020; X. Liu et al. 2020; Sounderajah et al. 2025; Tejani et al. 2024; Collins et al. 2024; Moons et al. 2025). No pooled effect estimates or formal risk‐of‐bias scores were calculated. Selection was purposive, and no claim of exhaustive coverage is made; this limitation is considered in Section 9. The translational stages and their permissible interpretations are summarized in Table 1. Figure 1 summarizes the translational‐evidence pathway and the evaluative gates applied at different stages.

**TABLE 1:** Translational‐evidence pathway used for interpretation.

Level	Evidence stage	Minimum supporting features	Permissible interpretation
1	Development/proof of concept	Retrospective development set; patient‐level split reported; appropriate task metrics	Technical feasibility only
2	Independent external validation	Geographically, temporally, or technically independent cohort; calibration and subgroup results where relevant	Portability to the tested setting
3	Prospective clinical or workflow evaluation	Prospective use with predefined outcomes and clinician comparator or workflow endpoint	Clinical performance or operational utility
4	Regulated or deployed system	Authorization for a defined indication plus real‐world monitoring	Use is limited to the authorized intended purpose
5	Patient‐level benefit	Controlled evidence of improved treatment, functional outcome, safety, or resource use	Clinical effectiveness for the studied population and workflow

*Note*: The stages represent translational position rather than an ordinal hierarchy of study quality; regulatory authorization or deployment may occur before or independently of prospective demonstration of clinical benefit.

**FIGURE 1:**
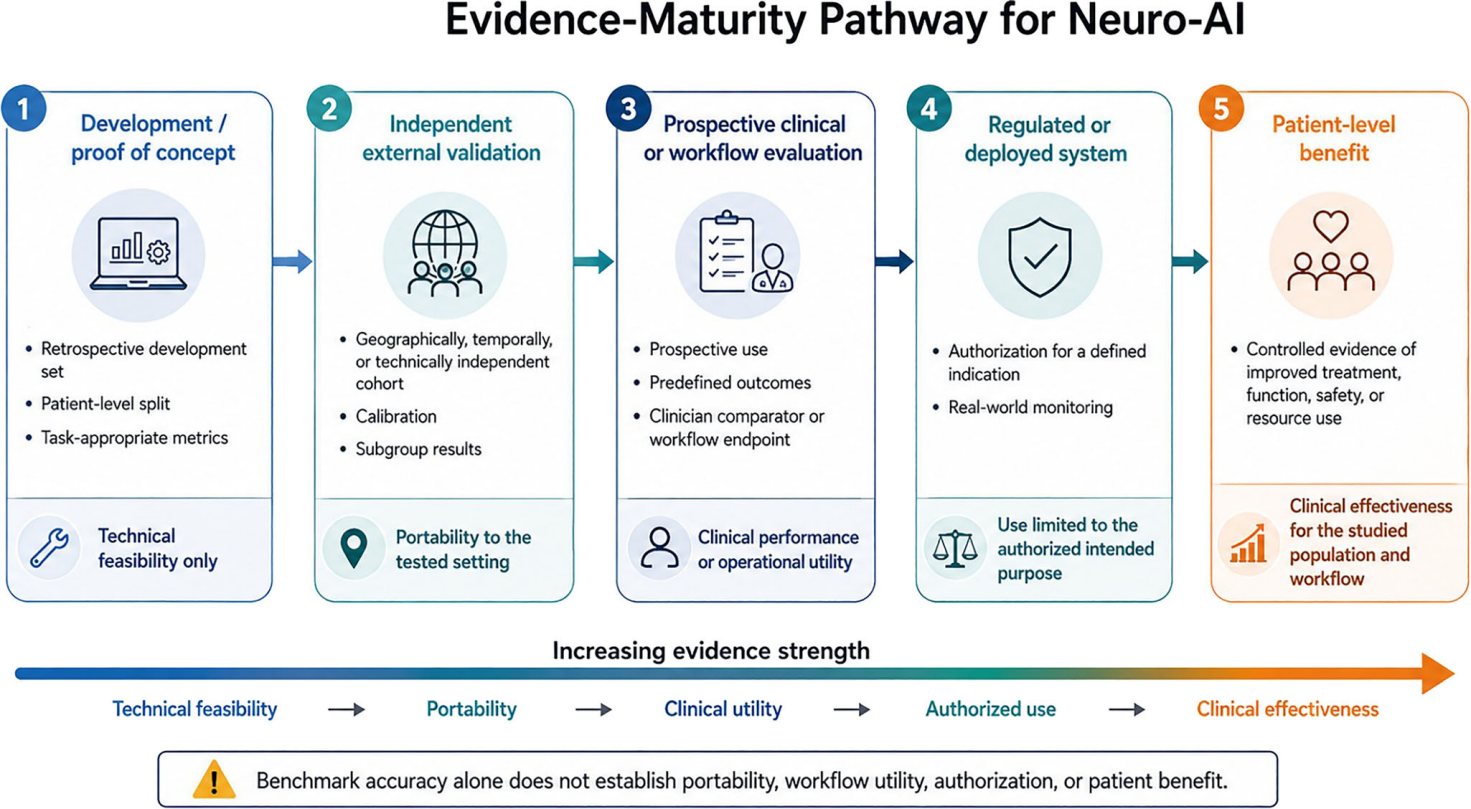
Translational‐evidence pathway for neuro‐AI. The pathway illustrates major translational milestones rather than a mandatory linear hierarchy; regulatory authorization or deployment may occur at different points relative to prospective clinical evaluation.

Seventeen primary studies were purposively selected for detailed comparison in Table 2. Selection was designed to illustrate variation in translational position across the six prespecified neurological domains rather than to identify the highest performing models or constitute an exhaustive sample. Priority was given to studies that represented one or more clinically informative translational features, including independent external validation, clinically relevant comparators, prospective or workflow evaluation, regulatory or deployed use, or assessment of patient‐level outcomes. Technical proof‐of‐concept studies were retained when they illustrated an important contrast between benchmark performance and clinical evidence. Where multiple reports evaluated the same cohort, system, or clinical task, the most complete or translationally informative report was prioritized. Accordingly, Table 2 should be interpreted as a purposive cross‐domain comparison rather than a representative epidemiological sample of the neuro‐AI literature.

**TABLE 2:** Representative studies illustrating differences in translational position.

Domain/task	Study and population	Input/model	Validation and key result	Evidence interpretation and limitation
Stroke workflow	243 thrombectomy‐treated patients; 4 centers (Martinez‐Gutierrez et al. 2023)	Automated CTA LVO detection plus secure team communication	Stepped‐wedge cluster‐randomized trial; 11‐min reduction in thrombectomy initiation	Level 3 workflow evidence; patient‐outcome effect not established
Stroke segmentation	ISLES 2022 n=250 plus independent 80‐patient set (Pacal et al. 2025)	DWI; FA‐UNet	Independent test Dice 0.868	External technical validation; no treatment or workflow outcome
AD/MCI prognosis	153 development trajectories and 741 ADNI validation trajectories (Fogel et al. 2026)	Routine clinical variables; regression ML	External MMSE MAE 2.19 points	Level 2; prospective decision impact not tested
PD speech screening	*n* = 1306, including 392 PD (Adnan et al. 2025)	Speech embeddings; multimodel fusion	Random split AUROC 0.889; unseen clinical sets 0.821 and 0.784	External decline demonstrates domain shift; not a stand‐alone diagnosis
PD smartwatch phenotyping	5159 tasks from 469 individuals (Varghese et al. 2024)	Bilateral inertial sensors plus questionnaire; stacked ML	Cross‐validated balanced accuracy 91.2% PD vs. controls; 72.4% PD vs. differential diagnoses	Internal validation; clinically realistic comparator reduces performance
Epilepsy/FCD detection	40 patients and 67 controls (Ding et al. 2025)	MR fingerprinting plus nnU‐Net	Leave‐one‐out validation; 80% sensitivity and 1.7 false positives/patient	Promising presurgical assistance; small cohort and no external site
MS differential diagnosis	322 MS/mimic and 84 prodromal cases (Wynen et al. 2026)	Central vein sign, cortical lesions, rim lesions; ML	Two out‐of‐distribution tests; simplified model balanced accuracy 97.2% and 92.6%	Level 2 multicenter evidence; retrospective and not yet workflow‐tested
Brain‐tumor classification	3064 public contrast‐enhanced MRI images (Ozger et al. 2026)	Five CNNs and five vision transformers	Unified benchmark; 96.7% CNN and 96.4% transformer accuracy	Image benchmark only; patient‐level separation and external clinical utility remain uncertain

*Note*: Translational stage describes where an application sits along the pathway and does not constitute a formal assessment of study quality or risk of bias. A complete structured extraction matrix is provided in Table S1.

## 3 Disease‐Specific Clinical Evidence

### 3.1 Stroke

Acute stroke is the clearest example of a neurological workflow in which minutes matter. AI systems are used for large‐vessel occlusion (LVO) and intracranial hemorrhage detection, Alberta Stroke Program Early CT Score (ASPECTS) estimation, perfusion analysis, team notification, and outcome prediction (Wolcott and English 2024; Saver 2006; Martinez‐Gutierrez et al. 2023; Powers et al. 2019). These tasks differ clinically: an alerting tool is judged by sensitivity, false alerts, and time‐to‐treatment, whereas segmentation and prognosis require spatial agreement, calibration, and decision‐curve or outcome evaluation.

The strongest current evidence concerns workflow rather than automated diagnosis in isolation. In a stepped‐wedge cluster‐randomized trial of 243 thrombectomy‐treated patients across four comprehensive stroke centers, automated computed tomography angiography (CTA)‐based LVO detection with secure team communication reduced time to thrombectomy initiation by 11 min (Martinez‐Gutierrez et al. 2023). Conversely, a prospective intracranial hemorrhage triage study did not improve radiology turnaround time, showing that algorithmic accuracy does not guarantee operational benefit (Savage et al. 2024).

External evaluations of e‐ASPECTS and commercial perfusion platforms show clinically useful agreement with experts, but systematic differences remain across software packages, scanners, lesion size, posterior‐circulation disease, and image quality (Mair et al. 2022; Kobeissi et al. 2023; Pisani et al. 2023; Bushnaq et al. 2024). Accordingly, outputs from competing platforms should not be treated as interchangeable, and local validation is necessary when thresholds influence thrombolysis or thrombectomy decisions.

Prognostic models that combine clinical and imaging data can discriminate 90‐day functional outcomes, but discrimination alone is insufficient. The MR PREDICTS@24 h model reported C‐statistics of 0.89–0.91, and fused deep‐learning models have approached expert performance (Chalos et al. 2023; Y. Liu et al. 2024); however, calibration, transportability, treatment‐related confounding, and clinical net benefit require further evaluation before these tools guide individual treatment decisions.

Recent 2025–2026 studies extend stroke lesion segmentation through attention‐gated U‐Nets, adaptive‐scale modules, gated bottlenecks, and comparative detector benchmarks (Kunduracioglu et al. 2026; Pacal et al. 2025, 2026; Pacal and Kunduracioglu 2026; Ganie and Pacal 2026b; Ince et al. 2026). FA‐UNet, for example, was trained on Ischemic Stroke Lesion Segmentation (ISLES) 2022 data and tested on an independent cohort of 80 patients, reporting a Dice coefficient of 0.868 (Pacal et al. 2025). These advances improve technical benchmarking, but lesion overlap on diffusion‐weighted imaging (DWI) or CT is not equivalent to faster reperfusion, better functional outcome, or safe autonomous triage.

Several products, including Rapid NCCT Stroke, Brainomix e‐ASPECTS, Viz LVO, and Avicenna.AI CINA‐LVO, have received US regulatory clearance for defined decision‐support indications (US Food and Drug Administration 2023a, 2023b; US Food and Drug Administration 2022a, 2022b). Clearance establishes an authorized intended use; it does not establish superiority across all hospitals or patient groups. Post‐deployment monitoring should therefore assess false alerts, missed cases, treatment times, subgroup performance, and drift after scanner or protocol changes.

Overall, stroke has progressed furthest toward prospective workflow evaluation and regulated or deployed use, but evidence of patient‐level clinical benefit remains limited. The most defensible clinical claim is that selected AI‐enabled systems can improve specific workflow intervals when embedded in well‐designed stroke pathways; broader claims of improved neurological outcome require adequately powered controlled studies.

### 3.2 Dementia and AD

AD is a common cause of dementia and is characterized biologically by amyloid‐beta and tau pathology (Scheltens et al. 2021; McDade 2022; Yarns et al. 2022; Raji and Benzinger 2022). AI studies address differential diagnosis, mild cognitive impairment (MCI)‐to‐dementia progression, biomarker prediction, and longitudinal decline using MRI, PET, biofluids, cognitive testing, speech, and digital measures.

The evidence is heterogeneous. Many studies use repeatedly sampled public datasets such as the Alzheimer's Disease Neuroimaging Initiative (ADNI), where site overlap, repeated scans, and image‐level rather than patient‐level splitting can inflate apparent performance. External validation in a genuinely independent health system is therefore more informative than another internally cross‐validated benchmark.

#### 3.2.1 Classification and Biomarker Prediction

Classical ML models identified structural patterns such as hippocampal atrophy (Y. Zhang et al. 2015; Fabrizio et al. 2021), while DL models expanded feature extraction from three‐dimensional MRI and PET (Y. Zhang et al. 2021; Kim and Lee 2023; Apostolopoulos et al. 2022). Reported accuracies approaching 99% should not be interpreted as clinical diagnostic accuracy unless the class distribution, patient‐level split, external test cohort, confidence intervals, and comparison with specialist assessment are clearly reported. High accuracy in balanced case‐control datasets may fall substantially in memory‐clinic populations with mixed pathologies and uncertain diagnoses.

#### 3.2.2 Progression and Prognosis

Prediction of MCI progression and functional decline is more clinically demanding than case‐control classification because outcomes are time‐dependent and treatment, comorbidity, and missing follow‐up can introduce bias (Y. Zhang et al. 2021; Liss et al. 2021; Kale et al. 2024; Christodoulou et al. 2025). A 2026 study developed models using 153 routinely assessed 12‐month trajectories and externally validated cognitive decline on 741 ADNI trajectories; the external mean absolute error (MAE) for the Mini‐Mental State Examination (MMSE) score was 2.19 points (Fogel et al. 2026). This design is more informative for portability than high internal classification accuracy, although prospective clinical utility and decision impact remain untested.

#### 3.2.3 Explainability and Clinical Readiness

Saliency maps, attention weights, and Shapley additive explanation (SHAP) values can show which image regions or variables influence a prediction, but they do not prove causal reasoning, model correctness, or fairness (Taiyeb Khosroshahi et al. 2025; Lundberg and Lee 2017; Samek et al. 2020). Calibration, uncertainty estimates, and error analysis across disease stage, ancestry, education, scanner, and comorbidity may be more clinically useful than visually persuasive heatmaps.

Multimodal models can outperform single‐modality approaches when modalities are complete and harmonized, yet missing PET, biofluid, or neuropsychological data can make such systems impractical outside research cohorts (Kale et al. 2024; M. Wang et al. 2024; Battineni et al. 2024; R. Zhang et al. 2025). Models using routine clinical data may offer lower headline accuracy but greater accessibility and transportability.

No reviewed model should replace established clinical and biomarker criteria. Near‐term value lies in quantitative decision support, risk stratification, and trial enrichment, with clinicians retaining responsibility for diagnosis and communication. Prospective evaluation should test whether AI changes diagnosis timing, counseling, treatment eligibility, or patient outcomes without increasing inequity.

### 3.3 PD and Movement Disorders

#### 3.3.1 Motor, Speech, and Digital Phenotyping

PD is the second most common neurodegenerative disorder. Its heterogeneous motor and non‐motor manifestations, fluctuating medication response, and gradual progression make one‐time clinic ratings an incomplete representation of daily function (Kehnemouyi et al. 2024; Rovini et al. 2017; Sotirakis et al. 2023; Hendricks and Khasawneh 2021).

Clinical scales such as the Movement Disorder Society Unified Parkinson's Disease Rating Scale remain essential, but inter‐rater variability and sparse sampling motivate objective complementary measurements. Video, speech, inertial sensors, keystroke tasks, breathing signals, and smartphones can quantify behavior; these devices become AI systems only when ML is used to derive a classification, prediction, or control policy.

Recent studies illustrate both promise and limitation. FastEval Parkinsonism reported acceptable agreement rates of 88.0% and 81.5% for left‐ and right‐hand video tasks (Y. Y. Yang, Ho et al. 2024), while deep models have extracted movement features without prespecified human measurements (J. Yang, Williams et al. 2024). Such results support measurement assistance, not autonomous diagnosis, because camera position, task adherence, disease mimics, medication state, and demographic differences affect generalizability.

Speech models are becoming more robust. Adnan et al. (2025) analyzed 1306 participants, including 392 with PD, and reported an area under the receiver operating characteristic curve (AUROC) of 0.889 in a randomized split; performance decreased to 0.821 and 0.784 in two unseen clinical datasets. This decline is informative rather than discouraging: it demonstrates why independent testing is needed before remote screening claims are made.

The clinical role of these tools is most plausibly triage, longitudinal measurement, or enrichment for clinical trials. Diagnostic evaluation must still exclude atypical parkinsonism, medication effects, structural disease, and other causes of tremor or gait impairment.

#### 3.3.2 Digital Biomarkers and Longitudinal Monitoring

A digital biomarker is an objective, quantifiable physiological or behavioral measure collected by digital technology; it is not synonymous with a wearable device or an AI model (Biomarkers Definitions Working Group 2001; Byrom et al. 2018; Coravos et al. 2019; S. Y. Meng, Yun et al. 2024; Dockendorf et al. 2021). Validity requires reliable sensing, an interpretable clinical construct, repeatability, responsiveness to change, and evidence that the measure adds value beyond established assessment.

The open Parkinson's Disease Smartwatch dataset captured 5159 task recordings from 469 individuals. Cross‐validated models achieved 91.2% balanced accuracy for PD versus healthy controls but only 72.4% for PD versus differential diagnoses (Varghese et al. 2024). The latter comparison better resembles clinical practice and exposes spectrum bias hidden by easy case‐control discrimination.

Speech, sleep, facial expression, gait, reaction time, and passive non‐motor measures may enable home monitoring (S. Y. Meng, Yun et al. 2024; Varghese et al. 2024; Kwon et al. 2021; Skodda et al. 2013). However, adherence, sensor drift, device replacement, missingness, digital literacy, privacy, and unequal access can alter both inputs and outcomes. Longitudinal responsiveness and external validation are therefore more important than single‐session classification accuracy.

Evidence for patient benefit remains limited. Future trials should define whether digital measures guide medication adjustment, detect meaningful deterioration, reduce visits, improve quality of life, or merely produce additional data.

#### 3.3.3 AI‐assisted Deep Brain Stimulation

Deep brain stimulation (DBS) is an established treatment for appropriately selected patients with PD (Deuschl et al. 2006); it should not be described as an emerging therapy. AI is emerging within the DBS workflow for candidate selection, electrode localization, outcome prediction, biomarker detection, and adaptive parameter control (Oliveira et al. 2023; Inggas et al. 2024; Opri et al. 2020).

Adaptive DBS may use electrophysiological biomarkers and control algorithms to adjust stimulation (Oliveira et al. 2023). Supervised learning has been studied for localization and outcome prediction (Inggas et al. 2024), whereas reinforcement learning remains largely preclinical or simulation‐based (M. Lu et al. 2020). Closed‐loop stimulation is not automatically AI‐enabled: fixed thresholds and conventional control rules should be distinguished from models that learn or update from data.

The main translational challenges are small and selected cohorts, patient‐specific signals, limited cross‐device portability, safety constraints on autonomous adaptation, and the need for interpretable failure modes. Prospective comparisons with expert programming should measure symptom control, adverse effects, programming time, battery use, and clinician workload.

AI may improve DBS efficiency and personalization, but established multidisciplinary selection, surgical accuracy, and longitudinal clinical assessment remain the standard of care.

#### 3.3.4 Critical Appraisal

Across PD applications, models perform best for tightly controlled tasks and often decline when distinguishing PD from clinically similar disorders. Diverse external cohorts, standardized medication‐state reporting, longitudinal evaluation, and patient‐centered outcomes are needed before remote or multimodal systems can be used as diagnostic substitutes.

The most mature near‐term use is measurement support: objectively quantifying change between visits while preserving clinical interpretation and patient choice.

### 3.4 Epilepsy

#### 3.4.1 Seizure Detection, Forecasting, and Automated EEG

Epilepsy affects more than 70 million people and remains difficult to monitor because seizures may be unwitnessed, nocturnal, or unreported (Thijs et al. 2019; Beniczky et al. 2021; Fisher et al. 2012). Video‐EEG is the diagnostic reference standard for many questions but is impractical for continuous long‐term monitoring.

Wearable accelerometry, electrodermal activity, heart‐rate signals, limited‐channel EEG, and multimodal devices can support automated detection, particularly for generalized tonic‐clonic seizures (Beniczky et al. 2013, 2021; Faust et al. 2025; T. Y. Ding et al. 2024). Evidence should be reported by seizure type because sensitivity for convulsive events cannot be generalized to focal impaired‐awareness, absence, or subclinical electrographic seizures.

Forecasting is a distinct and harder task. It requires prospective, time‐respecting validation, individualized baselines, clinically acceptable false‐alarm rates, and evaluation of whether warnings change behavior or outcomes. Randomly splitting adjacent windows from the same patient can create leakage and overstate performance.

Studies of wearable systems remain relatively small and selected. Faust et al. (2025) evaluated 28 patients under video‐EEG supervision, and T. Y. Ding et al. (2024) studied nocturnal physiological features in 78 patients. These studies support feasibility but not population‐wide seizure forecasting.

Near‐term clinical value lies in objective seizure counting and safety alerts for defined seizure types. Broader forecasting claims require long‐duration, patient‐independent, multicenter evaluation.

#### 3.4.2 Automated EEG Interpretation

EEG remains central to epilepsy diagnosis and monitoring, but interpretation is time‐consuming and subject to interobserver variability (Pillai and Sperling 2006; Benbadis and Lin 2008). DL can assist with interictal epileptiform discharge detection, seizure detection, and record prioritization (Da Silva Lourenço et al. 2021b; Nhu et al. 2022), although performance depends strongly on montage, age, indication, artifacts, and prevalence.

Da Silva Lourenço et al. compared a previously trained network with seven experts. The network achieved 82.5% sensitivity at 99% specificity with fewer than 0.2 false detections per minute, outperforming a commercial comparator for individual discharge detection (Tjepkema‐Cloostermans et al. 2025; Da Silva Lourenço et al. 2021a). This represents clinically relevant validation, but single‐center data and expert disagreement still limit a universal ground truth.

A 2025 meta‐analysis reported pooled sensitivity of 0.96, specificity of 0.97, and AUROC of 0.99 across 93 datasets (Bai et al. 2025). These pooled values should be interpreted cautiously because heterogeneous populations, sampling units, class balance, internal validation, and possible patient‐level leakage can produce optimistic summary estimates.

Automated EEG should therefore be positioned as a second reader, triage tool, or quantitative assistant. Prospective studies must test reading time, false‐alert burden, missed clinically important patterns, clinician‐AI disagreement, and downstream treatment decisions.

#### 3.4.3 Imaging and Presurgical Localization

AI may assist detection of focal cortical dysplasia (FCD) (Severino et al. 2020; Walger et al. 2023) and integration of MRI, PET, EEG, and MEG for the localization of the epileptogenic zone in drug‐resistant epilepsy (S. Zhang, Zhuang et al. 2024; Říha et al. 2022). These are high‐value applications because subtle lesions may be missed and complete localization is associated with surgical outcome.

Multicenter FCD detection studies demonstrate improved sensitivity over conventional morphometric approaches, but scanner variability, lesion annotation, template registration, and false‐positive clusters remain major obstacles (Walger et al. 2023; House et al. 2021; Spitzer et al. 2022; Yao et al. 2020). Dice scores should be interpreted alongside patient‐level sensitivity and false positives per patient.

A 2024 pediatric neural‐network U‐Net (nnU‐Net) study included 65 pathology‐confirmed FCD II cases and reported mean Dice 0.57 and patient‐level sensitivity 0.73 (S. Zhang, Zhuang et al. 2024). A 2025 magnetic resonance (MR)‐fingerprinting model used 40 patients and 67 controls with leave‐one‐out validation, reaching 80% sensitivity with 1.7 false positives per patient (Z. Ding et al. 2025). These results are promising for lesion discovery but remain below the evidence required for autonomous surgical targeting.

Electric and magnetic source imaging provide complementary localization information, yet their performance varies with electrode density, source orientation, recording duration, and reference standard (Pellegrino et al. 2018; Coito et al. 2019; Ebersole and Ebersole 2010; Baumgartner 2004). AI can combine these modalities, but prospective comparison with multidisciplinary epilepsy‐surgery decisions is essential.

#### 3.4.4 Treatment Selection, Neurostimulation, and Outcome Prediction

About one‐third of patients have drug‐resistant epilepsy, and surgery or ablation may provide seizure freedom when a resectable epileptogenic zone is identified (Oldham et al. 2015; Hader et al. 2013; Jobst et al. 2020). Prediction models can support counseling, but they should not replace individualized multidisciplinary assessment.

The Cleveland Clinic nomograms were developed in 846 patients and validated in 604; discrimination in the external cohort was modest (concordance 0.60–0.61) despite acceptable visual calibration (Jehi et al. 2015). This illustrates why simple, externally validated clinical models may be more informative than complex internally validated models with higher apparent accuracy.

Peri‐ictal scalp‐EEG models have reported high accuracy for postoperative seizure‐outcome prediction (Sheikh et al. 2024), and a 2025 meta‐analysis reported pooled sensitivity and specificity of 0.84 with AUROC of 0.91 (Pereira Da Silva et al. 2025). Heterogeneity, retrospective design, treatment selection, and limited calibration prevent direct use as treatment rules.

High‐frequency oscillations (HFOs) are candidate biomarkers of epileptogenic tissue, not universally reliable markers (Jacobs et al. 2008; Gunnarsdottir et al. 2022; A. Li et al. 2018; Spring et al. 2020). Detection thresholds, recording hardware, sleep state, artifacts, and spatial sampling differ across studies, and resection of HFO‐generating tissue has not produced uniform outcome benefit.

Responsive neurostimulation is an established closed‐loop therapeutic platform, but current clinical systems are not synonymous with self‐learning AI (Inaji et al. 2020; Jarosiewicz and Morrell 2021; Ma and Rao 2018; Herbozo Contreras et al. 2024). Adaptive models may eventually personalize detection and stimulation, yet safety, latency, on‐device computation, cybersecurity, and controlled clinical evaluation remain prerequisites.

Across epilepsy, the most mature applications are constrained detection and second‐reader support. Forecasting, surgical‐outcome prediction, and adaptive neurostimulation remain investigational and should be described according to their validation stage.

### 3.5 Multiple Sclerosis

MS is a heterogeneous inflammatory and neurodegenerative disease that requires integration of clinical findings, MRI, cerebrospinal‐fluid or serum biomarkers, optical coherence tomography, cognitive assessment, and patient‐reported outcomes (McGinley and Goldschmidt 2021; Rao et al. 2020). AI applications include lesion segmentation, detection of new or enhancing lesions, differential diagnosis, progression prediction, treatment‐response modeling, and remote monitoring (Naji et al. 2023; Afzal et al. 2022).

Automated MRI segmentation can reduce manual workload and improve quantitative follow‐up, but models remain sensitive to scanner, field strength, sequence, lesion burden, and annotation protocol (Bonacchi et al. 2022; Rocca et al. 2024; Reinhardt et al. 2024; Vasileiadis et al. 2018). Lesion count alone is an incomplete prognostic marker; gray‐matter injury, brain atrophy, spinal‐cord disease, and clinical trajectory must be considered.

Reports of 96% accuracy for active‐versus‐inactive lesion classification without gadolinium are hypothesis‐generating unless the number of patients, patient‐level split, external test set, and prevalence are explicit (Rovira and Auger 2022; Tomassini et al. 2020; Shekari et al. 2024; Y. Peng et al. 2021). Image‐ or lesion‐level sampling can allow scans from the same patient to appear in training and testing, inflating performance.

A 2026 multicenter study combined the central vein sign, cortical lesions, and paramagnetic rim lesions in 322 patients with MS or mimics and 84 prodromal cases (Wynen et al. 2026). Simplified models retained balanced accuracy of 97.2% and 92.6% in two out‐of‐distribution test sets, supporting portability; nevertheless, the study was retrospective and prospective effect on misdiagnosis or treatment is not yet established.

Treatment‐response and progression models using clinical, imaging, and molecular variables remain less mature (Yperman et al. 2020; Amin et al. 2024). Commercial volumetric tools and curated lesion datasets improve technical standardization but do not establish clinical utility (Mendelsohn et al. 2023; Muslim et al. 2022). Remote gait and activity measures face similar problems of adherence, device variability, and limited prospective validation.

Clinical translation in MS requires longitudinal multicenter datasets, scanner‐aware validation, calibration, explicit missing‐data handling, and comparison with contemporary diagnostic criteria. Models should support, not bypass, neurological assessment and serial imaging review.

### 3.6 Neuro‐Oncology

AI in neuro‐oncology spans tumor detection, segmentation, grading, molecular prediction, digital pathology, surgical planning, treatment‐response assessment, and recurrence prediction (Ostrom et al. 2021; Wesseling and Capper 2018; Y. Yang et al. 2021; Sarker 2021). Each task has a different reference standard and clinical consequence; classification of a curated MRI dataset should not be conflated with molecular diagnosis or operative guidance.

Segmentation models based on U‐Net variants, convolutional neural networks (CNNs), and transformers can quantify enhancing tumor, non‐enhancing core, and edema (Y. Yang et al. 2021; C. Zhang et al. 2019; Khan et al. 2023). Public challenges such as the Brain Tumor Segmentation (BraTS) challenge have accelerated comparison, but repeated use of the same benchmarks, preprocessing pipelines, and hidden test sets can lead to benchmark overfitting (Dorfner et al. 2025; Bakas et al. 2019).

Radiogenomic models attempt to infer isocitrate dehydrogenase mutation, 1p/19q codeletion, methylation status, and other molecular features from imaging (F. Li, Li et al. 2025; Kha et al. 2021). Very high internal AUROC values are not substitutes for tissue diagnosis. Model evaluation should include independent institutions, contemporary World Health Organization classifications, confidence intervals, calibration, and clinically relevant indeterminate categories.

Recent benchmark studies illustrate rapid technical progress. A 2026 comparison of 10 CNN and vision‐transformer architectures used 3064 contrast‐enhanced MRI images and reported 96.7% accuracy for Inception‐v4 and 96.4% for PiT‐Base (Ozger et al. 2026). DiSCNet compared 71 architectures and reported 99.2% four‐class accuracy (Ganie and Pacal 2026a). Because these studies used public image datasets, patient‐level independence, scanner diversity, spectrum of mimics, and external clinical validation determine whether headline accuracy is transferable.

Digital pathology models can standardize region detection and grading, but whole‐slide performance is influenced by staining, scanning, institution, tumor sampling, and evolving molecular taxonomy (Acs et al. 2020; Baxi et al. 2022; Kather et al. 2019; Elazab et al. 2024). Pathology remains the diagnostic reference standard, and AI should be evaluated as an assistive reader with error analysis across rare subtypes.

For surgery, AI may assist preoperative segmentation, tract or vascular visualization, and intraoperative navigation; it is not an essential or autonomous surgical tool (Cè et al. 2023; S. Lu et al. 2019). Claims of improved extent of resection, neurological preservation, or survival require prospective controlled evidence rather than geometric segmentation alone.

Neuro‐oncology currently contains abundant technical proof‐of‐concept studies and strong shared benchmarks, but fewer externally validated, prospective, workflow, or outcome studies. Multicenter validation and linkage to molecular, treatment, and outcome data are the priorities for clinical translation.

## 4 Cross‐Cutting Modalities and Clinical Tasks

### 4.1 Neuroimaging, Radiomics, and Multi‐Omics

Neuroimaging AI supports segmentation, detection, classification, radiomics, radiogenomics, and prognosis across stroke, dementia, epilepsy, MS, and neuro‐oncology (Kale et al. 2024; Huang and Shu 2025; Brahma and Vimal 2024; Bacon et al. 2024). The shared technical challenge is distribution shift caused by scanner vendor, field strength, reconstruction, protocol, site, and patient case mix.

Multimodal models integrate imaging with clinical, electrophysiological, genomic, proteomic, or metabolomic data (Latifi‐Navid et al. 2025; L. Meng, Jin et al. 2024; Galldiks et al. 2020; Cüce et al. 2025). They may improve discrimination, but often select patients with complete high‐cost data and therefore risk lower applicability. Performance should be compared with a clinically available baseline, not only with another experimental model.

For high‐dimensional multi‐omics, sample size is frequently small relative to feature count. Feature selection performed before data splitting, batch effects, repeated measures, and outcome leakage can produce optimistic results. External testing, locked preprocessing, calibration, and transparent missing‐data strategies are essential.

### 4.2 Electrophysiology and Automated Signal Processing

EEG and related signals are time series with artifacts, nonstationarity, and marked patient variability (Ille et al. 2024; Golmohammadi et al. 2019). Conventional filtering, template matching, and spectral analysis should be distinguished from models that learn representations from data. DL and transformer models can detect events or prioritize records (Da Silva Lourenço et al. 2021b; Tjepkema‐Cloostermans et al. 2025; Bai et al. 2025), but a high segment‐level AUROC does not necessarily translate to accurate patient‐level diagnosis.

Evaluation must use patient‐independent and time‐respecting splits, report event‐based sensitivity and false alarms per hour, and test portability across ages, montages, devices, and institutions. For closed‐loop use, latency, power consumption, failure detection, and safe fallback behavior are as important as classification accuracy.

### 4.3 Natural Language Processing, LLMs, and Foundation Models

Natural language processing (NLP) extracts phenotypes, diagnoses, functional status, and trial eligibility from unstructured clinical text (Y. Wang et al. 2025; Simon et al. 2025; Nerella et al. 2024; Ballard et al. 2024). LLMs can additionally summarize, generate, retrieve, and interact, but their generative behavior creates risks of hallucination, citation fabrication, omission, and automation bias (Singhal et al. 2023; Goh et al. 2024; G. Zhang, Jin et al. 2024; H. Li, Fu et al. 2025).

Early neurological applications include patient education, speech‐derived phenotyping, report assistance, and evidence retrieval (Singhal et al. 2023; Goh et al. 2024; G. Zhang, Jin et al. 2024; H. Li, Fu et al. 2025). A randomized study found no significant improvement in clinician diagnostic accuracy with LLM assistance, emphasizing that fluent output is not proof of clinical benefit (Goh et al. 2024).

Foundation models and vision‐language systems may reduce task‐specific annotation requirements, but they can inherit undocumented training bias and fail silently under domain shift (Paschali et al. 2025). Neurology‐specific evaluation should use locked tasks, external institutions, calibrated uncertainty, human‐factors testing, and audit logs. Retrieval augmentation and clinician review reduce, but do not eliminate, factual error.

### 4.4 Digital Biomarkers, Decision Support, and Closed‐Loop Systems

Wearables collect data, digital biomarkers summarize clinically meaningful aspects of those data, decision‐support systems recommend or prioritize actions, and closed‐loop devices sense and intervene. A system may contain one, several, or none of these components, so the terms should not be used interchangeably.

Clinical decision‐support systems can improve timeliness or adherence when recommendations are specific, actionable, and well integrated. Poorly designed alerts produce fatigue, interruption, and overreliance (Yeung et al. 2023; Pradhan et al. 2024; AbuAlrob and Mesraoua 2024; Hussain et al. 2019; Miller et al. 2025). Closed‐loop neurostimulation adds additional safety requirements because model error can directly alter therapy.

Evaluation should therefore include usability, time burden, override behavior, alert frequency, clinician disagreement, cybersecurity, and unintended consequences in addition to discrimination. Human oversight must be active and appropriately informed, not merely a nominal approval step. Figure 2 summarizes how neurological data, AI tasks, clinical roles, and lifecycle safeguards connect in practice.

**FIGURE 2:**
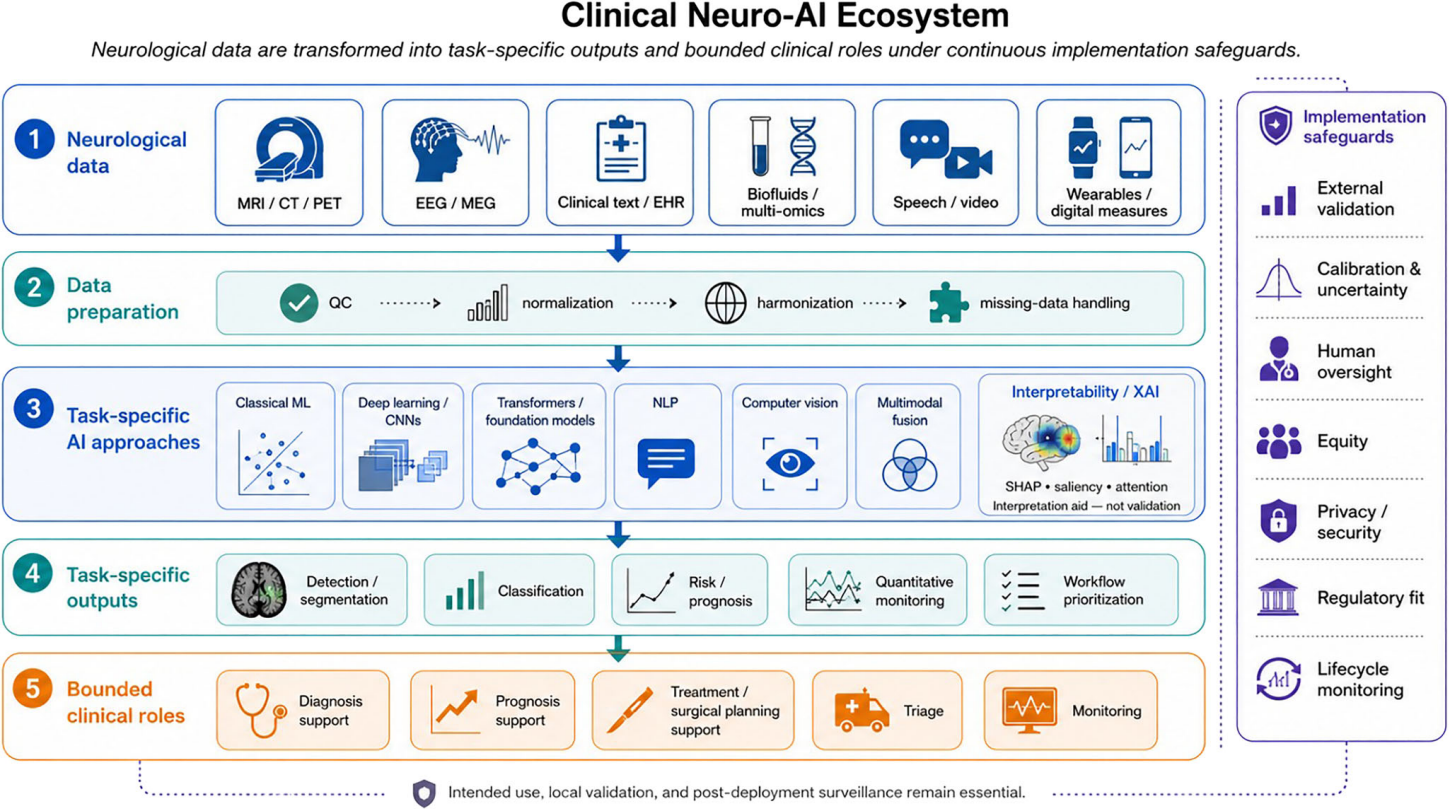
Clinical neuro‐AI ecosystem. Overview of multimodal neurological data, AI approaches, clinical outputs, and key implementation safeguards.

## 5 Emerging Architectures and Data Strategies (2022–2026)

### 5.1 Multimodal and Foundation Models

Multimodal systems combine imaging, EEG, clinical text, biofluids, genomics, or behavior through early, intermediate, or late fusion (Battineni et al. 2024; Amine and Mourad 2025; Zheng et al. 2024; Mazzotti 2024). Their advantage is the ability to represent complementary disease mechanisms; their weakness is dependence on synchronized, complete, and harmonized inputs that may not exist in routine care.

Foundation models use large‐scale pretraining to support multiple downstream tasks. In neurology and neuroradiology, their potential includes image interpretation, report generation, representation learning, and low‐label adaptation (Paschali et al. 2025). Current evidence is predominantly retrospective and task‐specific; broad capability claims require external testing across institutions, modalities, and patient groups.

A rigorous comparator should include both a clinically available baseline and simpler unimodal models. Added complexity is justified only when it improves calibration, transportability, workflow, or patient outcomes rather than a small benchmark metric.

### 5.2 FL, Privacy‐Preserving AI, and Open Data

FL trains models across sites without centralizing raw data. FeTS, MedPerf, OpenFL, and related initiatives demonstrate technical feasibility for multi‐institutional benchmarking and brain‐tumor or MS segmentation (Pati et al. 2022; Rehman et al. 2023; Yahiaoui et al. 2024; D. Liu et al. 2023). FL reduces raw‐data movement but does not by itself guarantee privacy, fairness, or generalization.

Sites may differ in labels, prevalence, hardware, and data quality; federated models can therefore reproduce or amplify heterogeneity. Secure aggregation, differential privacy, governance agreements, attack resistance, and site‐specific performance reporting remain necessary.

Open datasets such as ADNI, The Cancer Imaging Archive (TCIA), BraTS, the Temple University Hospital electroencephalography (TUH EEG) Corpus, the Children's Hospital Boston–Massachusetts Institute of Technology (CHB‐MIT) Scalp EEG Database, and OpenNeuro support reproducibility by allowing independent groups to evaluate models on the same data (Dorfner et al. 2025; Kiessner et al. 2023; Markiewicz et al. 2021; Bouchard et al. 2025). However, repeated benchmark use, incomplete demographic documentation, data leakage, and narrow acquisition settings can create a false sense of generalizability.

Reproducible neuro‐AI requires versioned data and code, explicit patient‐level splits, locked preprocessing, model cards, dataset documentation, and reporting of failed or negative results. Open data and federated collaboration are complementary strategies rather than substitutes.

The key translational shift is from maximizing performance on a familiar benchmark to demonstrating stability across changing clinical environments.

## 6 From Model Performance to Clinical Evidence

Reported metrics should be interpreted within a translational‐evidence pathway comprising technical proof of concept, independent external validation, prospective clinical or workflow evaluation, regulatory authorization or real‐world deployment, and demonstrated patient‐level benefit. These stages should not be interpreted as a strict evidence hierarchy or as necessarily sequential. In particular, regulatory authorization or deployment may occur without prospective demonstration of clinical benefit, while an investigational system may demonstrate prospective benefit before widespread deployment. The framework is therefore intended to describe where an application sits along the translational pathway rather than to provide an ordinal ranking of evidentiary strength.

Translational stage should also be distinguished from study quality. Studies occupying the same stage may differ substantially in risk of bias, sample size, patient representativeness, comparator quality, calibration, external validity, statistical precision, and completeness of reporting. These dimensions were therefore considered separately when interpreting individual studies.

### 6.1 Validation, Calibration, and Clinical Comparators

Random train‐test splits are inadequate when multiple images, windows, visits, or lesions come from the same patient. Patient‐level separation is the minimum standard; temporal, geographic, and technical external validation provide stronger tests of transportability.

Discrimination metrics such as accuracy and AUROC are prevalence‐sensitive in their clinical interpretation and can hide poor minority‐class performance. Studies should report sensitivity, specificity, predictive values, precision‐recall measures, confidence intervals, calibration‐in‐the‐large, calibration slope, and decision utility appropriate to the task (Rainio et al. 2024; Van den Goorbergh et al. 2022; Riley et al. 2024).

Clinical comparators should reflect intended use. An assistive model should be compared with clinicians working both with and without the tool; a triage model should measure time and false‐alert burden; a prognostic model should be compared with standard clinical scores and assessed for incremental net benefit.

Subgroup evaluation by age, sex, ancestry, language, socioeconomic context, disease stage, site, and device should be prespecified where relevant. Small subgroup counts create imprecise estimates and must not be interpreted as proof of fairness.

### 6.2 Deployment, Regulation, and Post‐Market Monitoring

Prospective evaluation should measure workflow, usability, override behavior, safety, resource use, and patient‐centered outcomes. DECIDE‐AI, SPIRIT‐AI, CONSORT‐AI, TRIPOD+AI, STARD‐AI, and CLAIM provide stage‐specific reporting frameworks (Vasey et al. 2022; Rivera et al. 2020; X. Liu et al. 2020; Sounderajah et al. 2025; Tejani et al. 2024; Collins et al. 2024).

Regulatory authorization applies to a specified intended use, population, input, and operating environment (International Medical Device Regulators Forum 2025a, 2025b; International Medical Device Regulators Forum 2013). Model updates, new scanners, different prevalence, or altered workflows can change performance and may require revalidation. Predetermined change‐control plans and lifecycle monitoring are therefore central to adaptive AI (US Food and Drug Administration 2024).

Post‐market surveillance should include drift detection, calibration checks, subgroup auditing, incident reporting, cybersecurity, and a defined rollback or human‐only pathway. Deployment is the start of evidence generation, not the endpoint.

### 6.3 Technical Updates Published in 2025–2026

The most recent literature includes a 91‐study systematic review of acute‐stroke imaging (Kunduracioglu et al. 2026), together with controlled DWI detector comparisons, adaptive segmentation architectures, and compute‐efficient brain‐tumor classifiers (Pacal et al. 2025, 2026; Pacal and Kunduracioglu 2026b; Ganie and Pacal 2026b; Ince et al. 2026; Ozger et al. 2026; Ganie and Pacal 2026a). These publications are relevant to architecture selection and reproducibility but occupy a different translational position from prospective workflow studies or regulated products.

For example, FA‐UNet included an independent 80‐patient DWI test set, whereas ASMM‐Net and several related models focused on public benchmark datasets (Pacal et al. 2025; Pacal and Kunduracioglu 2026). GBA‐Net extended evaluation across CT and DWI, while DiSCNet and the 2026 CNN‐versus‐transformer benchmark emphasized accuracy and computational efficiency for tumor classification (Ganie and Pacal 2026b; Ozger et al. 2026; Ganie and Pacal 2026a).

The critical comparison is therefore not which architecture reports the highest metric, but whether patient‐level independence, external institutions, clinically representative mimics, calibration, prospective use, and effect on decisions or outcomes have been demonstrated. Table 3 summarizes selected recent technical updates and their evidence limits.

**TABLE 3:** Selected 2025–2026 technical updates and their evidence limits.

Study	Task and data	Reported contribution	Translational interpretation
Kunduracioglu et al. (2026)	Systematic review of 91 CT/MRI acute‐stroke studies	Compared segmentation, triage, and prognosis literature	Shows rapid growth but persistent heterogeneity and limited prospective validation
Ince et al. (2026)	DWI ischemic‐lesion localization	Controlled comparison of recent YOLO detectors	Lesion‐level benchmark; clinical triage and outcome utility remain untested
FA‐UNet, 2025 (Pacal et al. 2025)	ISLES 2022 *n* = 250 plus independent; *n* = 80 DWI test	Attention‐gated, computationally efficient segmentation	Useful external technical validation; no workflow or treatment evidence
ASMM‐Net, 2025 (Pacal and Kunduracioglu 2026)	ISLES 2022 DWI benchmark	Adaptive‐scale modulation for lesion segmentation	Public benchmark performance; needs independent multicenter testing
GBA‐Net, 2026 (Ganie and Pacal 2026b)	CT and DWI stroke segmentation datasets	Gated bottleneck and attention across modalities	Cross‐dataset technical evidence; clinical portability and calibration remain unknown
DiSCNet, 2026 (Ganie and Pacal 2026a)	Four‐class public brain‐tumor MRI classification	Compared with 71 CNN, transformer, and hybrid architectures	High accuracy does not establish patient‐level external clinical validity
Ozger et al. (2026)	3064 contrast‐enhanced MRI images	Unified comparison of 10 CNN/transformer models	Strong benchmark design; image provenance and patient‐level independence remain decisive

### 6.4 Open Science and Reproducibility

Public data, shared code, and reporting standards improve auditability, but reproducibility also requires disclosure of exclusions, preprocessing, hyperparameter selection, seed variation, missing data, label adjudication, and all tested outcomes (Dorfner et al. 2025; Kiessner et al. 2023; Markiewicz et al. 2021; Jwa and Poldrack 2022).

Leaderboards should prevent hidden test‐set reuse and report uncertainty, computational cost, and subgroup results. Independent replication is more informative than repeated small improvements on the same benchmark.

When data cannot be shared, executable code, synthetic test fixtures, model cards, federated evaluation, or secure external validation can still support reproducibility.

These practices are especially important in fast‐moving fields, where unavailable code and rapidly superseded architectures make published claims difficult to verify.

## 7 Limitations and Challenges of Neuro‐AI Evidence

### 7.1 Data Quality, Bias, and Dataset Shift

Labels in MRI, CT, EEG, and electronic health records may reflect expert disagreement, incomplete observation, coding practice, or weak proxy outcomes (Jwa and Poldrack 2022; Wei et al. 2024; J. Yang, Triendl et al. 2024). Multi‐rater adjudication, uncertainty‐aware labels, transparent relabeling, and sensitivity analyses are preferable to treating every annotation as error‐free ground truth.

Class imbalance is intrinsic to rare tumors, uncommon seizure types, posterior‐circulation stroke, and many safety events. Overall accuracy can obscure clinically important failures; predictive values, class‐specific sensitivity, calibration, and clinically weighted error analysis are required (Rainio et al. 2024; Van den Goorbergh et al. 2022).

Scanner, protocol, device, language, and site differences create nonbiological batch effects. Harmonization can reduce these differences but may remove genuine biological variation or fail at unseen sites (Marzi et al. 2024; Y. Liu and Yap 2024; van Nederpelt et al. 2024). External validation should therefore follow, not be replaced by, harmonization.

Algorithmic bias arises from who is represented, how disease is measured, which patients receive testing, and how missingness is handled. Fairness observed in development data may not persist after deployment under population or acquisition shift (Y. Yang, Zhang et al. 2024; Ktena et al. 2024).

Continuous monitoring is necessary because prevalence, workflows, software, and hardware change. A model can remain technically operational while becoming clinically miscalibrated.

### 7.2 Reproducibility and Generalizability

Many studies remain difficult to reproduce because code, preprocessing, patient‐level splits, and complete performance estimates are unavailable. Small external datasets also produce unstable calibration and subgroup estimates; sample‐size planning should target precision for discrimination, calibration, and net benefit rather than rely on rules of thumb alone (Riley et al. 2024).

Single‐center retrospective performance should be described as local evidence. Claims of generalizability require external sites, devices, disease spectra, and workflows, ideally followed by prospective evaluation.

Reporting frameworks improve transparency but cannot compensate for weak design. Adherence to TRIPOD+AI, STARD‐AI, CLAIM, and related guidance should be accompanied by appropriate sampling, locked analysis, and complete reporting of negative and subgroup results (Vasey et al. 2022; Rivera et al. 2020; X. Liu et al. 2020; Sounderajah et al. 2025; Tejani et al. 2024; Collins et al. 2024).

### 7.3 Clinical Validation and Deployment

Prospective and randomized evidence remains scarce relative to the volume of algorithm‐development studies (Han et al. 2024; Moosavi et al. 2024). Even among authorized products, adoption is concentrated, and real‐world effectiveness varies by setting (Wu et al. 2024).

Human–AI interaction can improve signal detection or reduce workload, but it can also create alert fatigue, automation bias, and deskilling. Cross‐specialty evidence of poorer unaided performance after routine AI exposure is cautionary, although direct transfer to neurology is unproven (Graafsma et al. 2024; Scott et al. 2024; Budzyn et al. 2025).

Safe deployment requires user‐centered design, training, clear responsibility, monitoring, and the ability to question or override outputs. Institutions need governance for procurement, validation, updates, incidents, and retirement of models.

## 8 Ethical, Legal, and Medico‐Legal Issues

### 8.1 Privacy, Consent, and Data Governance

Neurological data are difficult to anonymize. Structural neuroimaging can encode facial and anatomical identity, continuous EEG and wearable streams reveal behavior, genomics is intrinsically identifying, and clinical text may contain rare combinations of dates, locations, and diagnoses. Governance must therefore address re‐identification, secondary use, linkage, retention, and access rather than rely on de‐identification alone.

Consent should explain foreseeable secondary uses, commercial involvement, cross‐border transfer, model updating, and whether patients can withdraw future use. Data minimization, purpose limitation, role‐based access, encryption, FL, secure aggregation, and differential privacy may reduce risk but introduce accuracy, equity, and operational trade‐offs (International Medical Device Regulators Forum 2025b; European Parliament and Council 2024; Sheller et al. 2020; Wilkinson et al. 2016).

The General Data Protection Regulation and the EU AI Act establish overlapping obligations for personal data and high‐risk AI, while US and other jurisdictions distribute responsibility across privacy, medical‐device, malpractice, and health‐information rules (European Parliament and Council 2016, 2024; European Commission 2023). Multicenter projects require jurisdiction‐specific legal review and explicit data‐controller and processor responsibilities.

Data provenance should document the source, consent, exclusions, demographic composition, acquisition process, annotation, and permitted reuse of every dataset. Reuse of clinical recordings for new predictive objectives should not be assumed to be ethically equivalent to the original clinical purpose.

Privacy and reproducibility can coexist through governed access, federated evaluation, standardized documentation, and auditable analysis environments, but no technical method removes the need for institutional accountability.

### 8.2 Transparency, Accountability, and Clinician–AI Interaction

Transparency should match the decision. Clinicians need to know the intended use, target population, required inputs, contraindications, training and validation settings, calibration, subgroup performance, uncertainty, update policy, and known failure modes (Vasey et al. 2022; Collins et al. 2024; Health Data, Technology, and Interoperability: Certification Program Updates 2025). A saliency map alone does not meet this need (Lundberg and Lee 2017; Samek et al. 2020).

Responsibility must be allocated before deployment. Developers are accountable for design, testing, documentation, cybersecurity, and update control; institutions for procurement, local validation, monitoring, workflow design, and training, and clinicians are accountable for using the tool within its intended purpose while applying professional judgment. Responsibility cannot be displaced onto an opaque algorithm.

Human oversight should protect against both overreliance and reflexive rejection. Interfaces should make uncertainty and data quality visible, support appropriate override, and record clinician‐AI disagreement for quality review. Patients should be informed when AI materially contributes to high‐stakes decisions where disclosure is legally or ethically required.

### 8.3 Regulation, Liability, and Post‐Market Monitoring

AI used for diagnosis, prognosis, or treatment may qualify as Software as a Medical Device (International Medical Device Regulators Forum 2013). Regulatory expectations increasingly address data quality, human factors, cybersecurity, change management, and lifecycle monitoring (International Medical Device Regulators Forum 2025a, 2025b). A research prototype, commercially marketed product, and authorized device must be labeled distinctly in scholarly discussion.

Predetermined change‐control plans can permit specified future modifications while preserving regulatory oversight (US Food and Drug Administration 2024). Adaptive updates outside the authorized plan, off‐label use, and local retraining may alter liability and require new validation.

Medico‐legal risk arises from erroneous outputs, failure to act, automation bias, poor integration, unavailable explanations, and unmonitored drift. Institutions should define who reviews alerts, response times, downtime procedures, audit retention, escalation, and incident investigation. Authorization does not eliminate negligence or product‐liability questions.

### 8.4 Equity, Underrepresentation, and Access

Underrepresentation is not limited to race or ethnicity. Older adults, children, people with disability, rural populations, low‐resource settings, minority languages, uncommon disease phenotypes, and patients unable to complete digital tasks may all be absent from development data (Uwishema and Boon 2025; Rajpurkar et al. 2022; Koçak et al. 2024; Lamem et al. 2025).

Bias can enter through referral, access to imaging or specialist diagnosis, label quality, missing modalities, device ownership, and loss to follow‐up. Models may also infer sensitive attributes from images or signals even when those variables are removed (Koçak et al. 2024; Celi et al. 2022).

Fairness assessment should report subgroup sample sizes, uncertainty, calibration, false‐positive and false‐negative consequences, and intersectional performance. Equal aggregate accuracy is not sufficient when clinical harms differ across groups.

Mitigation includes representative recruitment, local validation, participatory design, low‐bandwidth or offline options, accessible interfaces, transparent thresholds, and continuous monitoring. Threshold changes should be clinically justified and should not conceal unequal data quality.

AI can widen access only if the surrounding services can respond. A screening alert without confirmatory testing, treatment capacity, referral pathways, or patient trust may increase anxiety and inequity rather than improve care.

## 9 Limitations of This Review

This review is narrative and purposive rather than systematic. Although the search strategy, sources, timeframe, eligibility criteria, and prioritization framework are reported, formal duplicate screening by independent reviewers, a PRISMA flow diagram, meta‐analysis, and study‐level risk‐of‐bias scoring were not performed. Selection bias and uneven representation of the literature therefore remain possible.

Publication bias may further favor positive or high‐performing AI models, while negative validation studies, failed external replications, and null workflow evaluations may be underrepresented. Much of the available evidence also remains retrospective and internally validated, limiting conclusions about real‐world transportability.

The six disease domains were chosen for depth and translational relevance and do not represent all of neurology. Heterogeneity across clinical tasks, datasets, metrics, and validation designs prevents direct ranking of diseases or algorithms. Commercial and regulatory information can also change after publication.

AI literature evolves rapidly, and some 2025–2026 studies discussed here are early technical benchmarks without prospective validation. The review therefore emphasizes evidence maturity and limitations rather than treating recent publication or high performance as proof of clinical utility.

The translational framework should not be interpreted as a formal hierarchy of study quality. Studies classified at the same translational stage may differ substantially in risk of bias, sample size, comparator quality, calibration, representativeness, and external validity, and regulatory or deployment status does not itself establish clinical effectiveness.

## 10 Future Directions

Future neuro‐AI studies should begin with an explicit clinical decision, intended user, comparator, and harm model. Development should use patient‐level separation, representative multicenter data, prespecified analysis, calibration, uncertainty estimation, subgroup evaluation, and transparent reporting. External validation should precede prospective workflow studies, and prospective studies should measure decisions, time, safety, resource use, function, quality of life, and other outcomes relevant to patients.

Technical priorities include robust multimodal models that tolerate missing inputs, foundation models evaluated under domain shift, privacy‐preserving collaboration, low‐resource deployment, interpretable uncertainty, and continuous post‐market monitoring. Progress should be measured by reproducibility and clinical benefit, not only by increasingly complex architectures or small benchmark gains.

## 11 Conclusion

AI has credible and growing roles in neurological image analysis, electrophysiology, quantitative monitoring, and workflow support, but maturity varies markedly by task. The strongest evidence supports selected assistive systems evaluated in external or prospective settings; much of the literature remains retrospective and benchmark‐centered. Safe clinical translation requires calibration, multicenter validation, clinician comparison, prospective utility, clear regulatory status, equity assessment, and lifecycle surveillance. AI should augment neurological judgment within accountable care systems, not be presented as an autonomous substitute for clinicians or as clinically transformative before patient‐level benefit is demonstrated.

## Author Contributions

**Anwar Zahran**: supervision, project administration, Writing – original draft, methodology, validation, Writing – review and editing. **Raya Fuqha**: writing – original draft, writing – review and editing, project administration. **Aya Milhem**: writing – original draft, writing – review and editing. **Orabi Hajjeh**: writing – review and editing, writing – original draft, project administration. **Hossam Salameh**: writing – review and editing, writing – original draft. **Mohammed Abubaha**: writing – original draft, writing – review and editing. **Waseem Shehadeh**: writing – original draft, writing – review and editing. **Hammam Jallad**: writing – original draft, writing – review and editing, visualization. **Mohammad Bdair**: writing – original draft, writing – review and editing, methodology. **Issa Snoubar**: writing – original draft, writing – review and editing. **Omar Abu–khazneh**: writing – original draft, writing – review and editing. **Bara Abubaha**: writing – review and editing, writing – original draft. **Ameer Awashra**: writing – original draft, writing – review and editing. **Aseel Eid**: writing – original draft, writing – review and editing. **Ahmad Mohammad**: writing – original draft, writing – review and editing, supervision. **Ahmad R. Awwad**: writing – original draft, writing – review and editing, supervision. All authors contributed to the interpretation of the evidence, critically reviewed the manuscript for important intellectual content, approved the final version, and accept accountability for the work.

## Funding

The authors have nothing to report.

## Ethics Statement

The authors have nothing to report.

## Conflicts of Interest

The authors declare no conflicts of interest.

## Supporting information




**Supplementary Material**: brb371873‐sup‐0001‐SuppMat.docx

## Data Availability

The authors have nothing to report.
