# Supplementary material for: Artificial Intelligence in Neurology: A Critical Narrative Review of Clinical Evidence, Translation, and Responsible Implementation
Source: Brain Behav. 2026 Nov 6;16(11):e71873. doi: 10.1002/brb3.71873 (PMC13741994; doi:10.1002/brb3.71873)
Supplement: Supplementary file 1 [file BRB3-16-e71873-s001.docx]

Supplementary Materials

*Artificial Intelligence in Neurology: A Critical Narrative Review of Clinical Evidence, Translation, and Responsible Implementation*

# Supplementary Methods S1. Search execution and study selection

The review was designed as a structured narrative review rather than a systematic review. PubMed/MEDLINE and IEEE Xplore were searched for English-language publications dated from 1 January 2022 through 31 July 2026. Final electronic searches and a targeted update were completed on 7 August 2026. The 2022 start date was chosen a priori to foreground contemporary model classes, validation practices, and regulatory expectations; older reports were retained only when required to establish a clinical tool, historical benchmark, or methodological concept.

Candidate records were reviewed by title and abstract and then in full text when potentially eligible. The review did not use independent duplicate screening. Duplicate and overlapping reports were identified using title, digital object identifier, cohort, and system name; the most complete or clinically mature report was prioritized. Because selection was purposive rather than exhaustive, a PRISMA-style count of retrieved and excluded records was not prospectively maintained and was not retrospectively fabricated.

## PubMed/MEDLINE strategy

(("artificial intelligence"[tiab] OR "machine learning"[tiab] OR "deep learning"[tiab] OR "neural network*"[tiab] OR "large language model*"[tiab] OR "foundation model*"[tiab] OR "federated learning"[tiab] OR "explainable AI"[tiab] OR radiomic*[tiab] OR "digital biomarker*"[tiab]) AND (neurolog*[tiab] OR stroke[tiab] OR dementia[tiab] OR Alzheimer*[tiab] OR Parkinson*[tiab] OR "movement disorder*"[tiab] OR epilep*[tiab] OR seizure*[tiab] OR "multiple sclerosis"[tiab] OR "brain tumor*"[tiab] OR glioma*[tiab] OR neuroimaging[tiab] OR electroencephalogra*[tiab] OR EEG[tiab]) AND ("2022/01/01"[Date - Publication] : "2026/07/31"[Date - Publication]) AND English[lang])

## IEEE Xplore strategy

(("All Metadata":"artificial intelligence" OR "All Metadata":"machine learning" OR "All Metadata":"deep learning" OR "All Metadata":"neural network" OR "All Metadata":"large language model" OR "All Metadata":"foundation model" OR "All Metadata":"federated learning" OR "All Metadata":"explainable AI" OR "All Metadata":"digital biomarker") AND ("All Metadata":neurology OR "All Metadata":stroke OR "All Metadata":dementia OR "All Metadata":Alzheimer OR "All Metadata":Parkinson OR "All Metadata":epilepsy OR "All Metadata":seizure OR "All Metadata":"multiple sclerosis" OR "All Metadata":"brain tumor" OR "All Metadata":glioma OR "All Metadata":neuroimaging OR "All Metadata":EEG)); filters: publication years 2022–2026; English language.

## Other sources

Primary regulatory and governance materials were sought on the websites of the US Food and Drug Administration, European Union, World Health Organization, and International Medical Device Regulators Forum. Reference lists of key reviews and primary studies were examined for landmark or clinically mature reports. These targeted searches were used for authoritative context and were not treated as an exhaustive grey-literature search.

# Supplementary Table S1. Structured extraction matrix for representative neuro-AI evidence

Reference numbers correspond to the main manuscript. NR indicates that the field was not reported or was not extracted in the narrative-review summary; it does not prove that the information is absent from the full publication. Regulatory authorization must not be inferred from publication alone.

| **Disease / clinical task** | **Study / reference** | **N / dataset** | **Data / model** | **Validation / patient separation** | **External validation** | **Comparator** | **Performance / calibration / CI** | **Explainability** | **Regulatory / deployment** | **Main limitation / evidence level** |
| --- | --- | --- | --- | --- | --- | --- | --- | --- | --- | --- |
| Stroke / thrombectomy workflow | Martinez-Gutierrez et al., 2023 (27) | 243 thrombectomy-treated patients; 4 centers | CTA; automated LVO detection plus secure team communication | Stepped-wedge cluster-randomized prospective workflow trial; patient-level clinical episodes | Multicenter prospective evaluation; independence from algorithm development not stated | Control periods under the stepped-wedge design | 11-minute reduction in thrombectomy initiation; CI/calibration not reported in the review summary | NR | Research workflow evaluation; authorization not inferred | Patient-level outcome effect not established; evidence level 3 |
| Stroke / intracranial hemorrhage triage | Savage et al., 2024 (29) | N not extracted in the narrative review | Noncontrast head CT; AI triage/prioritization | Prospective clinical evaluation; patient-level workflow outcome | Prospective setting; external relation to development data NR | Routine radiology workflow | No improvement in radiology turnaround time; CI/calibration NR | NR | Regulatory status NR | Demonstrates that algorithmic performance may not improve workflow; evidence level 3 |
| Stroke / ASPECTS estimation | Mair et al., 2022 (30) | N not extracted in the narrative review | Brain CT; commercial e-ASPECTS software | External clinical validation | Independent clinical evaluation | Expert/consensus readings | Clinically useful agreement, with systematic software- and case-dependent differences; detailed CI/calibration NR | NR | Commercial system; authorization and deployment effects not assessed by this study | Scanner, lesion-size, image-quality, and posterior-circulation limitations; evidence level 2 |
| Stroke / lesion segmentation | Pacal et al., 2025; FA-UNet (37) | ISLES 2022 n≈250; independent test n=80 | DWI; attention-gated FA-UNet | Independent test cohort; patient-level separation reported for the external set | Yes—independent 80-patient technical test set | Ground-truth lesion masks / benchmark methods | Dice 0.868 on the independent test set; CI/calibration NR | Attention architecture; no clinical explanation method | Research benchmark; not deployed | No workflow or treatment outcome; evidence level 2 technical validation |
| AD/MCI / cognitive prognosis | Fogel et al., 2026 (58) | 153 development trajectories; 741 ADNI validation trajectories | Routine clinical variables; regression ML | Development and external trajectory validation; longitudinal observations | Yes—separate ADNI validation trajectories | Observed longitudinal cognitive scores | External MMSE MAE 2.19 points; prospective decision impact and CI/calibration details NR | NR | Research model; not deployed | Clinical utility untested; evidence level 2 |
| Parkinson disease / speech screening | Adnan et al., 2025 (71) | n=1,306; 392 with Parkinson disease | Speech embeddings; multimodel fusion | Random development split followed by testing in two unseen clinical datasets | Yes—two unseen clinical datasets | Clinical diagnostic labels; no clinician comparator reported | AUROC 0.889 internally; 0.821 and 0.784 in unseen datasets; calibration/CI NR | NR | Research screening model; not a stand-alone diagnosis | Marked domain shift in external datasets; evidence level 2 |
| Parkinson disease / smartwatch phenotyping | Varghese et al., 2024 (77) | 5,159 tasks from 469 individuals | Bilateral inertial sensors plus questionnaire; stacked ML | Cross-validation; patient-level independence not specified in the narrative summary | No external site | Healthy controls and clinically realistic differential diagnoses | Balanced accuracy 91.2% versus controls and 72.4% versus differential diagnoses; CI/calibration NR | NR | Research dataset/model | Internal validation and spectrum effects; evidence level 1 |
| Epilepsy / automated EEG | Tjepkema-Cloostermans et al., 2025 (95–96) | Previously trained network; 7 expert readers; patient N NR in review summary | Clinical EEG; deep neural network | Validation of a previously trained model in a clinical dataset | Clinical validation reported; single-center generalizability remains limited | Seven experts and a commercial comparator | Sensitivity 82.5% at 99% specificity; <0.2 false detections/min; CI/calibration NR | NR | Research/clinical evaluation; authorization NR | Expert disagreement and single-center data; evidence level 2 |
| Epilepsy / pediatric FCD detection | Yao et al., 2024 (100) | 65 pathology-confirmed FCD II cases | Structural MRI; nnU-Net | Study-cohort evaluation; patient-level sensitivity reported | No external site reported | Pathology-confirmed cases / lesion masks | Mean Dice 0.57; patient sensitivity 0.73; CI/calibration NR | Segmentation map only | Research model | Small selected cohort; evidence level 1 |

# Supplementary Table S1. Structured extraction matrix (continued)

| **Disease / clinical task** | **Study / reference** | **N / dataset** | **Data / model** | **Validation / patient separation** | **External validation** | **Comparator** | **Performance / calibration / CI** | **Explainability** | **Regulatory / deployment** | **Main limitation / evidence level** |
| --- | --- | --- | --- | --- | --- | --- | --- | --- | --- | --- |
| Epilepsy / FCD detection | Ding et al., 2025 (105) | 40 patients; 67 controls | MR fingerprinting; nnU-Net | Leave-one-out validation; patient-level cases/controls | No external site | Clinical labels and lesion annotations | Sensitivity 80%; 1.7 false positives/patient; CI/calibration NR | Segmentation map only | Research model | Small cohort and no external validation; evidence level 1 |
| Epilepsy surgery / outcome prediction | Jehi et al., 2015 (113) | 846 development; 604 external validation patients | Clinical variables; nomograms | Separate development and external validation cohorts | Yes—external cohort | Observed postoperative seizure outcomes | External concordance 0.60–0.61; acceptable visual calibration | Interpretable clinical nomogram | Clinical prediction model; regulatory status not applicable/NR | Modest discrimination despite validation; evidence level 2 |
| Multiple sclerosis / differential diagnosis | Wynen et al., 2026 (136) | 322 MS/mimic cases; 84 prodromal cases | Central vein sign, cortical lesions, rim lesions; ML | Two out-of-distribution tests; patient-level cohorts | Yes—two out-of-distribution test sets | Clinical diagnostic categories including mimics | Balanced accuracy 97.2% and 92.6%; CI/calibration NR | Simplified feature model | Research model; not workflow-tested | Retrospective evidence; evidence level 2 |
| Neuro-oncology / tumor classification | Ozger et al., 2026 (151) | 3,064 public contrast-enhanced MRI images; class distribution NR | Five CNNs and five vision transformers | Unified public benchmark; patient-level independence uncertain | No independent clinical site | Ten architecture families / public labels | 96.7% CNN and 96.4% transformer accuracy; CI/calibration NR | NR | Research benchmark | Image provenance and patient independence uncertain; evidence level 1 |
| Acute stroke / evidence synthesis | Kunduracioglu et al., 2026 (36) | 91 CT/MRI studies | Segmentation, triage, and prognosis models | Systematic review across heterogeneous designs | Varied across included studies | Varied across included studies | No single pooled clinical-effect estimate used in this review | Varied/NR | Included research and technical evidence; deployment varied/NR | Persistent heterogeneity and limited prospective validation; synthesis across evidence levels |
| Stroke / DWI lesion localization | Ince et al., 2026 (41) | Dataset N NR in narrative summary | DWI; recent YOLO detectors | Controlled detector benchmark; patient-level separation NR | No independent clinical site reported | Multiple YOLO detector architectures | Lesion-level benchmark results; calibration/CI NR | Detector localization output | Research benchmark | Clinical triage and outcome utility untested; evidence level 1 |
| Stroke / multimodal segmentation | Ganie and Pacal, 2026; GBA-Net (40) | CT and DWI datasets; N NR in narrative summary | Gated bottleneck/attention model | Cross-dataset technical evaluation; patient-level separation NR | Cross-dataset, not clinical external validation | Benchmark segmentation methods | Technical performance reported; calibration/CI NR | Attention architecture; clinical explanation NR | Research benchmark | Clinical portability and calibration unknown; evidence level 1–2 technical |
| Neuro-oncology / four-class MRI classification | Ganie and Pacal, 2026; DiSCNet (152) | Public brain-tumor MRI data; N and class distribution NR in narrative summary | DiSCNet; CNN/transformer comparison | Public benchmark; patient-level independence uncertain | No external clinical site | 71 CNN, transformer, and hybrid architectures | Accuracy 99.2%; CI/calibration NR | NR | Research benchmark | High accuracy does not establish clinical validity; evidence level 1 |
